# The Coal-Tar Derivatives*Read before the Atlanta Society of Medicine, February 19, 1895.

**Published:** 1895-03

**Authors:** J. W. Duncan

**Affiliations:** Atlanta, Ga.


### THE COAL-TAR DERIVATIVES.*

BY J. W. DUNCAN, M.D.,

ATLANTA, GA.

The coal-tar derivatives and their combinations are so numerous that the mere naming of them all would require as much time as I would be expected to consume in a short introductory paper this evening.

I will not attempt to give a complete list of them. Some of them are somewhat similar in their chemical composition, and quite so in their physical and therapeutical properties. While others are quite dissimilar. They are solids, semi-solids, and liquids.

*Antipyrin and Antifebrin,* I class as twin sisters, as I commenced their use about the same time, giving them in doses as directed by the literature on them at that time, and I soon discovered that if I continued to use them in heroic doses, I would kill some of my patients, so I reduced the dose to a minimum one, and got good results without the cyanosis and other alarming symptoms. I seldom give either one now, without combining with it some agent to modify its effects.

*Sulphonal* is an excellent hypnotic, but it should be used with discretion, and should not be continued too long for fear of producing sulphonism.

*Tolypyrin* seems by the history given by P. Guttmann to have an effect very much like Antipyrin.

---

* Read before the Atlanta Society of Medicine, February 19, 1895.

*Trional*, I have used only in one case. Did not get any results in the ordinary dose.

*Hypnal*, a combination of Chloral and Antipyrin, is recommended in chorea, delirium tremens, and insomnia. I have not tried it.

*Phediuretin* acts as a diuretic probably by its effect on the central nervous system.

*Phenacetin* is diaphoretic, antipyretic, analgesic, and antiseptic, and may be used locally as well as constitutionally. It is one of the leading members of this large family in my practice.

*Phenocoll Hydrochlorate* is said to be antipyretic, analgesic, antiperiodic, etc.

*Salacetol*, introduced by P. Fritsch, is intended as a substitute for salol, and if it be better than salol, it is a good one.

*Salipyrin* is a Salicylate of Antipyrin, and has nothing to specially recommend it.

*Salocol*, according to P. Schering has several good features. He says it is antipyretic, antineuralgic, and antirheumatic, certain in its action and presenting little danger. The dose is large, from 15 to 30 grains, several times a day.

*Salol*.—To my mind, this is one of the most useful of the list, being antiseptic, analgesic, antirheumatic, antipyretic, antifermentative, etc. I have used it extensively in diarrhea, dysentery, rheumatism, typhoid fever, intestinal fermentation, tympanites, etc.

*Salophen*, Koch says, acts promptly in rheumatism, and he thinks it is better than salicylate of soda or salol. The dose is large (one and a half drachms daily).

*Steresol* is a new varnish-like substance, used for dressing sores, etc.

*Formanilid* is the homologue below acetanilid. It is claimed that it will cause complete anesthesia and that it is more efficacious than cocaine, the anesthesia lasting longer.

*Aniline*—methylene blue, is used internally and hypodermically in doses of one-half to seven grains two or three times a day, in rheumatism, neuralgia, and to reduce temperature.

*Analgene* is used for the same purposes as Antipyrin. It is tasteless, but causes the urine to be red, which may alarm the patient.

*Antinervine* is a new remedy for colds and rheumatism.

*Antipyrin* has been in use for some years and is recognized as an antipyretic, antiseptic, analgesic, etc.

*Asaprol* is said to be especially useful in rheumatism. It is said to be preferable to salol or salicylate of soda, as it does not cause tinnitus aurium and it is not contraindicated in albuminuria.[1]

*Beta-Naphthol*, from which the above is derived, is highly praised for its antiseptic properties in gastric and intestinal affections. I have used it only in one case and that was a case of consumption. I used it hypodermatically. It seemed to control the fever. No bad effects followed, except some soreness at point of injection.

*Benzosol* is highly praised in diabetes mellitus.

*Bismuth-naphthol-hydrated* is said to be a nontoxic antiseptic and will arrest fermentation at once. Claimed to be useful in intestinal disorders, etc.

*Bismuth-tribromphenol* is a new antiseptic, and is one of the late specifics against cholera.

*Bismuth-Beta-Naphthol* is praised for its effects in diarrhea.

There are several other combinations of the coal-tar derivatives with bismuth-phenates, etc.

*Carbolic Acid* is one that has been in general use for many years, and is still a favorite with us all. You are all familiar with its uses and virtues.

*Exalgin* relieves pain, and is said to be useful in chorea.

And what more shall I say, for time would fail me to speak of sulphaminol, tetrinol, antikamnia, monobrom-phenol, naphthalin, phenyl-hydrezin, phenosalyl, resorcin, thermodin, antitoxine, salophen, tolerine, trecrosal, trecresalimine, picric acid, saccharine, anthrarobin, asaprol, benzo guaiacol, betol, creasote, creolin, cresalol, gallanol, gallabromol, guaiacol, hydroquione, latrol, ichthyol, lysol, salicylic acid, phenylacetic, amido phenol, alumnol, sulphosalicylic, etc.

I will close by stating some conclusions from my clinical experience with the coal-tar derivatives. They are valuable additions to our therapeia. They are almost or quite indispensable to the surgeon or gynecologist, as well as to the general practitioner. When judiciously used in appropriate cases the results are good; but when given in heroic doses and continued for a long time they are as potent for evil.

The more active antipyretics of this class should not be relied upon to control hyperpyrexia in the continued fevers or in pneumonias, as we have other remedies which are equally as efficacious, and which are not so likely to produce injurious blood changes.

I have often observed the slow convalescence of patients recovering from fevers after the use of coal-tar derivatives, and I have reason to believe that relapses are more common also.

In the ephemeral fevers, catarrhal fever, neuralgias, rheumatism, la grippe, etc. (when not complicated with pneumonia), I have been very well satisfied with their effects. As a rule the dose need not be large and should not be continued for too long a time.

I have used creolin quite frequently and believe it to be an excellent antiseptic. I have tried it diluted as an enema in dysentery, but was not satisfied with its effects. It caused too much pain, and I failed to observe any curative action.

I have not used in my practice all the agents mentioned in this paper, and I doubt if I ever do, and I have only mentioned the names of a few of this prolific family. But I hope that I have said enough to elicit a full and free discussion.

(*See Society Reports.*)

